# Delineating effects of angiopoietin-2 inhibition on vascular permeability and inflammation in models of retinal neovascularization and ischemia/reperfusion

**DOI:** 10.3389/fncel.2023.1192464

**Published:** 2023-06-12

**Authors:** Jérémie Canonica, Richard Foxton, Marina Garcia Garrido, Cheng-Mao Lin, Sabine Uhles, Sumathi Shanmugam, David A. Antonetti, Steven F. Abcouwer, Peter D. Westenskow

**Affiliations:** ^1^Roche Pharma Research and Early Development, Roche Innovation Center, Basel, Switzerland; ^2^Department of Ophthalmology and Visual Sciences, Kellogg Eye Center, University of Michigan Medicine, Ann Arbor, MI, United States

**Keywords:** angiopoietin-2, choroidal neovascularization, diabetic macular edema, neovascular age-related macular degeneration, vascular endothelial growth factor-A

## Abstract

**Introduction:**

Clinical trials demonstrated that co-targeting angiopoietin-2 (Ang-2) and vascular endothelial growth factor (VEGF-A) with faricimab controls anatomic outcomes and maintains vision improvements, with strong durability, through 2 years in patients with neovascular age-related macular degeneration and diabetic macular edema. The mechanism(s) underlying these findings is incompletely understood and the specific role that Ang-2 inhibition plays requires further investigation.

**Methods:**

We examined the effects of single and dual Ang-2/VEGF-A inhibition in diseased vasculatures of JR5558 mice with spontaneous choroidal neovascularization (CNV) and in mice with retinal ischemia/reperfusion (I/R) injuries.

**Results:**

In JR5558 mice, Ang-2, VEGF-A, and dual Ang-2/VEGF-A inhibition reduced CNV area after 1 week; only dual Ang-2/VEGF-A inhibition decreased neovascular leakage. Only Ang-2 and dual Ang-2/VEGF-A inhibition maintained reductions after 5 weeks. Dual Ang-2/VEGF-A inhibition reduced macrophage/microglia accumulation around lesions after 1 week. Both Ang-2 and dual Ang-2/VEGF-A inhibition reduced macrophage/microglia accumulation around lesions after 5 weeks. In the retinal I/R injury model, dual Ang-2/VEGF-A inhibition was statistically significantly more effective than Ang-2 or VEGF-A inhibition alone in preventing retinal vascular leakage and neurodegeneration.

**Discussion:**

These data highlight the role of Ang-2 in dual Ang-2/VEGF-A inhibition and indicate that dual inhibition has complementary anti-inflammatory and neuroprotective effects, suggesting a mechanism for the durability and efficacy of faricimab in clinical trials.

## 1. Introduction

Retinal and choroidal vascular diseases, such as diabetic retinopathy, diabetic macular edema (DME), retinal vein occlusion, and neovascular age-related macular degeneration (nAMD), are among the leading causes of blindness and visual impairment globally (Laouri et al., 2011; Flaxman et al., 2017). A key driver of the pathology in these diseases is vascular leakage. In early-stage DME and retinal vein occlusion, this is primarily a result of damage to the retinal vasculature, whereas in the advanced stages of these diseases (including proliferative diabetic retinopathy), the growth of new, unstable blood vessels (neovascularization), which are characteristically leaky, plays an important role (Joussen et al., 2021). Neovascularization is the main driver of pathology in nAMD (Joussen et al., 2021). The edema resulting from vascular leakage has a detrimental effect on retinal architecture and, hence, on retinal function, and, ultimately, visual outcomes (Antonetti et al., 2021; Joussen et al., 2021).

Tissue ischemia leading to hypoxia and oxidative stress in nAMD, coupled with chronic hyperglycemia in diabetic retinopathy, mediates increased expression of vascular endothelial growth factor (VEGF)-A and pro-inflammatory cytokines, which stimulate increased vascular permeability and pathological neovascularization (Campochiaro, 2013; Joussen et al., 2021). VEGF-A and its receptors are required for developmental vasculogenesis and angiogenesis, whereas elevated VEGF-A expression drives vascular permeability and neovascularization in retinal and choroidal vascular diseases (Titchenell and Antonetti, 2013). Indeed, the introduction of intravitreal anti-VEGF agents has markedly improved outcomes in patients with these conditions in clinical trials over the last two decades (Martin et al., 2012; Nguyen et al., 2012; Busbee et al., 2013; Wells et al., 2016; Spooner et al., 2019; Adamis et al., 2020). However, maintaining vision outcomes with intravitreal anti-VEGF therapy requires regular injections, which are often not adhered to in real-world clinical practice, adding to the treatment burden (Varano et al., 2015; Ciulla et al., 2020, 2021). Further, the multi-factorial etiology of these diseases suggests that targeting additional pathways besides VEGF may facilitate greater vascular stability and prolonged disease remission, potentially contributing to increased treatment efficacy and durability compared with intravitreal anti-VEGF monotherapy (Grossniklaus et al., 2002; Rübsam et al., 2018; Joussen et al., 2021).

The angiopoietin (Ang)/tyrosine kinase with immunoglobulin (Ig) and epidermal growth factor homology domains (Tie) pathway plays an important role in vascular development and homeostasis. While Ang-1 activates the Tie2 receptor and promotes cell survival and vascular stability during development (Saharinen et al., 2017), Ang-2 acts predominately as an antagonist of Ang-1 and is often up-regulated under pathological conditions (Regula et al., 2016). Ang-2–related inhibition of Tie2 activation potentiates the action of VEGF, weakens the integrity of endothelial cell junctions, may promote detachment of pericytes, and is involved in the recruitment of inflammatory cells, all of which may underlie the vascular instability characteristics of retinal and choroidal vascular diseases (Campochiaro, 2015; Saharinen et al., 2017). In addition, independent of VEGF, Ang-2 may play a more direct underlying role in mediating vascular instability. Findings from preclinical studies suggest that Ang-2 can both alter Tie2 signaling and activate integrin signaling, leading to angiogenesis and endothelial de-stabilization (Felcht et al., 2012; Hakanpaa et al., 2015).

A synergistic role of Ang-2 and VEGF-A in vascular de-stabilization has previously been explored in JR5558 mice, a mouse model of spontaneous choroidal neovascularization (sCNV) in which mice develop subretinal neovascularization and fibrotic lesions resembling human nAMD (Nagai et al., 2014; Rossato et al., 2020). One week after JR5558 mice were treated with a bi-specific antibody targeting both Ang-2 and VEGF-A, there was a statistically significant reduction in choroidal neovascularization (CNV) lesion leakage area relative to IgG injection controls and to treatment with anti–Ang-2 or anti–VEGF-A alone (Regula et al., 2016; Foxton et al., 2019). Mice treated with the bi-specific antibody also showed a statistically significant reduction in photoreceptor apoptosis compared with mice treated with antibodies to Ang-2 or VEGF-A alone or controls (Regula et al., 2016; Foxton et al., 2019). In addition, retinal inflammation in JR5558 mice, assessed by evaluating the presence of ionized calcium binding adapter molecule 1 (Iba1)^+^ microglia/macrophages around CNV lesions, was statistically significantly reduced with dual Ang-2/VEGF-A inhibition compared with inhibition of Ang-2 or VEGF-A alone (Regula et al., 2016; Foxton et al., 2019). The reduction in inflammatory response with dual Ang-2/VEGF-A inhibition was also observed in an endotoxin-induced uveitis mouse model (Regula et al., 2016). These preclinical data suggest that Ang-2 and VEGF-A synergistically regulate vascular stability (vascular leakage, inflammation, and neovascularization) and that targeting both pathways in patients with retinal vascular diseases may be more beneficial than targeting VEGF-A alone. However, the longer-term effects of Ang-2 and dual Ang-2/VEGF-A inhibition in the mouse model of sCNV are yet to be evaluated.

Faricimab (at the time of writing, approved for the treatment of nAMD and DME in multiple countries worldwide as Vabysmo^®^) is a bi-specific antibody designed using Roche’s CrossMAb technology, which simultaneously neutralizes both Ang-2 and VEGF-A (Regula et al., 2016, 2019; Chakravarthy et al., 2017; Foxton et al., 2019). Following preclinical evaluation, dual Ang-2/VEGF-A inhibition with faricimab was explored in clinical trials in patients with DME and nAMD. The phase 2 BOULEVARD trial (NCT02699450) of faricimab in DME demonstrated superior vision outcomes with faricimab 6.0 mg compared with ranibizumab 0.3 mg in treatment-naïve patients with DME over 24 weeks (Sahni et al., 2019). In patients with nAMD, the 36-week AVENUE trial (NCT02484690) subsequently established the efficacy and safety of faricimab 6.0 mg compared with ranibizumab 0.5 mg (Sahni et al., 2020), whereas the 52-week STAIRWAY trial (NCT03038880) demonstrated sustained efficacy through extended durability of faricimab 6.0 mg dosed every 12 (Q12W) or 16 (Q16W) weeks, with similar vision and anatomic gains compared with monthly ranibizumab 0.5 mg (Khanani et al., 2020). Importantly, nearly two-thirds of patients were eligible for extended Q16W dosing in the STAIRWAY trial. More recently, 1-year results from the phase 3 YOSEMITE (NCT03622580) and RHINE (NCT03622593) DME trials demonstrated that faricimab 6.0 mg Q8W up to Q16W dosing was non-inferior for gains in best-corrected visual acuity and improved anatomic outcomes compared with aflibercept 2.0 mg Q8W (Wykoff et al., 2022). Likewise, 1-year results from the phase 3 TENAYA (NCT03823287) and LUCERNE (NCT03823300) trials of nAMD demonstrated that faricimab 6.0 mg up to Q16W was non-inferior to aflibercept 2.0 mg Q8W for gains in best-corrected visual acuity (Heier et al., 2022) and offered more rapid improvement in anatomical outcomes during the matched-dosing period (through week 12) (Cheung et al., 2022). In all these trials, faricimab demonstrated extended durability, with ∼ 50% of patients on faricimab Q16W dosing at 1 year. Faricimab was also well tolerated, with an acceptable safety profile (Heier et al., 2022; Wykoff et al., 2022). Subsequent 2-year findings for YOSEMITE/RHINE and TENAYA/LUCERNE showed that vision gains with faricimab 6.0 mg up to Q16W remained similar to those achieved with aflibercept 2.0 mg (Khanani et al., 2022; Lim et al., 2022) and that the improved anatomic outcomes observed in YOSEMITE/RHINE for faricimab compared with aflibercept were maintained through year 2 (Lim et al., 2022). Further, at year 2, a larger proportion of patients were on extended faricimab dosing compared with at year 1. Specifically, ∼ 80% of patients were on faricimab Q12W dosing and > 60% of patients were on faricimab Q16W dosing (Khanani et al., 2022; Lim et al., 2022). The mechanism(s) underlying these clinical findings are not completely understood, and the specific role that Ang-2 inhibition plays requires further investigation.

To further delineate the underlying effects of dual Ang-2/VEGF-A inhibition, including the extended durability findings and anatomical benefits observed in the phase 3 clinical trials of faricimab, we examined the therapeutic/preventative effects of single and dual Ang-2/VEGF-A inhibition using two different mouse models of retinal pathology. Specifically, we used the JR5558 mouse model of sCNV (Nagai et al., 2014) to evaluate the longer-term (5-week) vessel stabilization and anti-inflammatory effects. We also used the mouse retinal ischemia/reperfusion (I/R) injury model (Hartsock et al., 2016) to evaluate retinal vascular permeability and cell death.

## 2. Materials and methods

### 2.1. Animals

Mice were housed in temperature- (22 ± 1°C) and humidity- (57%) controlled rooms (automatic 12-h light/dark cycle) under pathogen-free conditions in groups of up to five in ventilated cages. Standard laboratory chow and tap water were supplied *ad libitum*. Studies on the mouse strain JR5558 with C57BL/6J background were performed using 7- (baseline) to 12- (5 weeks post treatment) week-old male and female animals. Studies on retinal I/R injury were performed using 10- to 11-week-old C57BL/6J male mice.

### 2.2. The JR5558 mouse model

The JR5558 genetic model (Hasegawa et al., 2014; Nagai et al., 2014) was discovered at The Jackson Laboratory, then established at the University College London, and was subsequently maintained and bred at Charles River Laboratories (Sulzfeld, Germany), from where it was sent to F. Hoffmann-La Roche Ltd., for *in vivo* experimentations.

The JR5558 experiments were designed as previously described (Regula et al., 2016; Foxton et al., 2019), but were extended to include a 5-week timepoint. JR5558 mice were given two intraperitoneal injections, separated by 1 week, of IgG control (10 mg/kg), anti–VEGF-A (5 mg/kg), or anti–Ang-2 (5 mg/kg) mouse cross-reactive tool antibodies or a bi-specific anti–Ang-2/anti–VEGF-A (10 mg/kg) antibody. An intraperitoneal route of administration was used for consistency with the previous JR5558 studies (Regula et al., 2016; Foxton et al., 2019) and to maintain good integrity of the eye, with a clear visual axis, given that a large portion of the data were generated from *in vivo* imaging. The 10 mg/kg anti–Ang-2/anti–VEGF-A antibody dose was selected based on our previously reported (Regula et al., 2016) anti-angiogenic dose-response findings for this antibody. Normal IgG antibodies, such as anti–VEGF-A and anti–Ang-2, have two binding sites for their specific targeted ligand, whereas the bi-specific anti–Ang-2/anti–VEGF-A antibody has one binding site for each target. Therefore, a 10-mg/kg concentration of anti–Ang-2/anti–VEGF-A antibody provides the same molar concentration of antigen binding sites for VEGF-A or Ang-2 as a 5-mg/kg concentration of the anti–VEGF-A and anti–Ang-2 antibodies. An untreated group of control JR5558 mice was also included. Neovascular leakage was analyzed *in vivo* by fluorescein angiography (FA) at baseline (day [D] 0) and at 1 week (D15) or 5 weeks (D43) after treatment to assess immediate and long-term treatment effects on CNV lesion activity, respectively. Blood-retinal barrier permeability was quantified by fluorophotometry 1 week after the last antibody dose. At the end of the study (D15 or D43), neovascularization and subretinal inflammatory cell infiltration were evaluated by immunohistochemistry on retinal pigment epithelium (RPE)/choroid flatmounts. Antibody treatment administration and the qualitative and quantitative analyses of results were made on a blinded basis.

### 2.3. Neovascular leakage analysis by FA

Fluorescein fundus angiography was carried out at baseline (D0) to equally assign JR5558 mice across experimental groups by sCNV lesion leakage area before treatment and 1 week (D15) or 5 weeks (D43) after treatment. Before fluorescein administration, mice were anesthetized by subcutaneous (SC) injection of a fentanyl (0.05 mg/kg), medetomidine (0.5 mg/kg), and midazolam (5 mg/kg) mixture, and eyes were dilated with 0.5% tropicamide. Once pupils were dilated, 3.2-mm plano contact lenses (Cantor & Nissel Ltd., Brackley, United Kingdom) were placed on the eyes, and 2% fluorescein sodium salt (10 mL/kg) was injected intraperitoneally. Mice were then placed in front of the Heidelberg Spectralis ophthalmic imaging system (Heidelberg Engineering, Heidelberg, Germany) for infrared (IR) and FA image acquisition. Both eyes underwent a complete analysis, with an initial central FA image captured 5 min after fluorescein injection and a subsequent six images to cover all sections of the eye. Following IR and FA imaging, anesthesia reversal was achieved by subcutaneous injection of a buprenorphine (0.2 mg/kg), atipamezole (2.5 mg/kg), and flumazenil (0.5 mg/kg) mixture. FA images from each individual eye were manually exported as JPEG files for analysis using Adobe Photoshop software (Adobe, Inc., San Jose, CA, United States). The number of lesion leakage areas per eye at baseline was counted using the Photoshop Count tool. For quantification of the lesion leakage area after treatment, areas of fluorescein leakage were selected for each eye with the Photoshop Lasso tool and expressed as pixel numbers for statistical analysis.

### 2.4. Fluorophotometry

*In vivo* quantitation of blood-retinal barrier permeability was performed by fluorophotometry (Fluorotron™ Master Laboratory Mouse Edition; OcuMetrics, Mountain View, CA, United States) 1 week (D15) after treatment. As per the FA procedure, JR5558 mice were anesthetized by subcutaneous injection of a fentanyl, medetomidine, and midazolam mixture. Eyes were then dilated using 0.5% tropicamide and contact lenses (Cantor & Nissel Ltd.) were placed to prevent eye drying and protect against cataract formation. For measurements of fluorescein concentration profiles in the vitreous ocular compartment and in the plasma, 1% fluorescein sodium salt (10 mL/kg) was administered by subcutaneous injection. Mice were then placed on a temperature-controlled (37.0 ± 1°C) stage, and their position was adjusted so that the eye being analyzed was aligned precisely and in parallel to the optic device. Data acquisition was started 45 min after fluorescein injection using 450- to 490-nm excitation and 520- to 600-nm emission for fluorescein detection. After scanning both eyes, tails were punctured for blood sampling (25 μL), and plasma was extracted by centrifugation (10,000 × *g* for 10 min) to correct for circulating fluorescein and, thus, account for potential administration differences. Plasma was then diluted 1:100 with phosphate-buffered saline (PBS) in a microcuvette, and fluorescein levels were measured using a cuvette holder provided with the fluorophotometer. Raw data for the vitreous compartment and plasma fluorophotometric measurements were analyzed and quantified using Microsoft Excel (Microsoft Corporation, Redmond, WA, United States) by calculating the average value from the five highest steps (axial distance) corresponding to the graphical peak for fluorescein concentration in the vitreous and plasma. Average fluorescein levels in the vitreous of JR5558 mice were corrected by the maximum plasma fluorescein values and expressed as a ratio on the final bar/scatter dot blot graph.

### 2.5. Immunohistochemistry analysis of RPE/choroid flatmounts

Subretinal inflammatory cell infiltration was detected by Iba1, CD45, and CD11b immunostaining and was evaluated by flatmounted RPE/choroid histology at 1 and 5 weeks after antibody treatment. Enucleated eyes were fixed with 4% paraformaldehyde solution for 2 h at room temperature (RT) and then transferred to PBS. The RPE/choroid complex was carefully separated from the retina and permeabilized for 2 h at RT in 3% Triton X-100 solution in PBS. After permeabilization, RPE/choroids were incubated overnight with the following primary antibodies in 0.3% Triton X-100 with 5% donkey serum in PBS: biotinylated isolectin B4 (lectin from *Bandeiraea simplicifolia*, 1:100, #L2140; Sigma-Aldrich, St. Louis, MO, United States), goat anti-Iba1 (1:200, #ab5076; Abcam, Waltham, MA, United States), rat anti-CD45 (1:200, #ab23910; Abcam), or rat anti-CD11b (1:200, #MCA711; Bio-Rad Laboratories, Hercules, CA, United States). The following day, the tissue was washed for 6 × 5 min with 0.3% Triton X-100 in PBS, followed by incubation for 2 h at RT with the following secondary antibodies in 0.3% Triton X-100 with 5% donkey serum in PBS: 1:100 fluorescein isothiocyanate (FITC)-conjugated DyLight 488 Streptavidin (1:100, #SA-5488; Vector Laboratories, Burlingame, CA, United States), donkey anti-goat IgG (1:200, Alexa Fluor 550, #A-21432; Thermo Fisher Scientific, Waltham, MA, United States), and anti-rat IgG (1:200, Alexa Fluor 647, #ab150155; Abcam). RPE/choroid tissues were then washed for 6 × 5 min in 0.3% Triton X-100 in PBS at RT before being flatmounted on Superfrost glass slides (Thermo Fisher Scientific) using Dako fluorescent mounting medium (#S3032; Dako, Glostrup, Denmark). Following RPE/choroid tissue immunostaining, isolectin B4, CD45, and CD11b signals were detected and acquired using an Olympus VS120 scanner equipped with a XM10 camera and a UPLSAPO 10 × /0.40 objective (Evident, Tokyo, Japan). Specific isolectin B4, Iba1, CD45, and CD11b signal areas were selected with the Lasso tool in Adobe Photoshop and areas of isolectin B4 signal or Iba1-, CD45-, and CD11b-positive cells number per RPE/choroid flatmount were quantified using ImageJ.

### 2.6. Retinal I/R injury mouse model

Two days before I/R injury, C57BL/6J mice were given intravitreal injections (1.4 μL/eye) of IgG control antibody (3 μg/μL) or anti–VEGF-A (anti–VEGF-A at 1.5 μg/μL and IgG control at 1.5 μg/μL), anti–Ang-2 (anti–Ang-2 at 1.5 μg/μL and IgG control at 1.5 μg/μL), or bi-specific anti–Ang-2/anti–VEGF-A (3 μg/μL). Two days after treatment, retinal ischemia was transiently induced by injection of saline into the anterior chamber to raise the intraocular pressure to 85–95 mmHg (measured *via* a TonoLab Rebound Tonometer; Icare Finland Oy, Vantaa, Finland), followed by natural reperfusion, as previously described (Abcouwer et al., 2021), except that the duration of ischemia was 75 min. Sham treatment comprised needle puncture only. Vascular permeability and ongoing cell death were assessed 48 h after I/R injury.

### 2.7. FITC-labeled BSA permeability assay

As a measure of retinal vascular permeability, retinal accumulation of fluorescein isothiocyanate–labeled bovine serum albumin (FITC-BSA) was measured at 48 h after I/R injury using a previously described method (Abcouwer et al., 2021). Briefly, the mice were given an intravenous injection of 200 mg/kg body weight of FITC-BSA (Sigma-Aldrich, St. Louis, MO, United States), which was allowed to circulate for 2 h before the retinal vasculature was flushed by transcardiac perfusion with PBS before retinal harvesting. Retinal FITC-BSA accumulation was quantified by fluorescence using a microplate spectrophotometer (FLUOstar Omega; BMG LABTECH, Ortenberg, Germany) and normalized to plasma FITC-BSA and retinal dry weight.

### 2.8. DNA fragmentation assay

A DNA fragmentation assay was used to evaluate ongoing retinal cell death 48 h after I/R injury using a previously described method (Abcouwer et al., 2021). Briefly, DNA fragmentation assay was carried out using Cell Death Detection ELISA PLUS (Roche Diagnostics, Basel, Switzerland) per the manufacturer’s instructions, and optical densities were detected using a microplate spectrophotometer (FLUOstar Omega) and normalized to retinal wet weight.

### 2.9. Blinding

All personnel involved in image acquisition, image analysis, and histology were blinded to treatment until raw data were fully processed. Only personnel involved in drug administration had information about the identity of the treatments the animals received.

### 2.10. Statistics

Data were compared between groups using one-way analysis of variance followed by Tukey’s multiple comparison test. Statistical significance was indicated by *P < 0.05, **P < 0.01, ***P < 0.001, and ****P < 0.0001. Statistical analyses were performed using Prism 7 (GraphPad Software, San Diego, CA, United States).

### 2.11. Study approval

Animal maintenance and experimental procedures were approved by the Federal Food Safety and Veterinary Office of Switzerland (reference BS-2734) and were conducted in strict adherence to the Swiss federal ordinance on animal protection and welfare, as well as according to the rules of the Association for Research in Vision and Ophthalmology Statement for the Use of Animals in Ophthalmic and Vision Research guidelines (European Directive 86/609/EEC) and the Roche Ethics Committee on Animal Welfare (JR5558 sCNV mouse model) or with approval of the University of Michigan Institutional Animal Care and Use Committee (retinal I/R injury mouse model).

## 3. Results

### 3.1. Dual Ang-2/VEGF-A inhibition reduces sCNV and lesion leakage area in JR5558 mice

#### 3.1.1. One week after treatment

**FIGURE 1:**
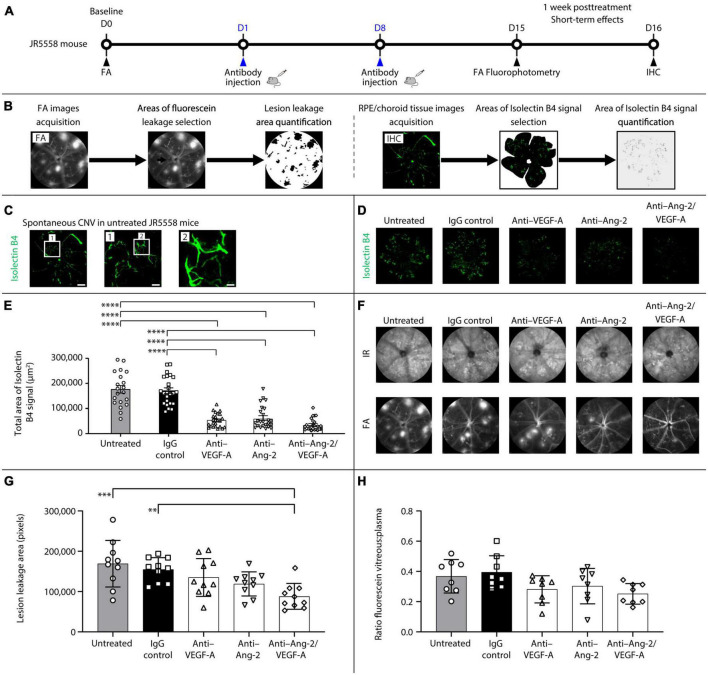
Dual angiopoietin-2 (Ang-2)/vascular endothelial growth factor-A (VEGF-A) inhibition reduces spontaneous choroidal neovascularization (CNV) and lesion leakage area in JR5558 mice. **(A)** General experimental protocol used to assess short-term treatments effects on limiting vascular leakage, neovascularization, and inflammation in the JR5558 mouse model. **(B)** Examples of lesion leakage area and total area of isolectin B4 signal quantification. **(C)** Representative isolectin B4 immunostaining images detected on retinal pigment epithelium (RPE)/choroid flatmounts from untreated JR5558 mice. Isolectin B4 is a marker for endothelial cells allowing visualization and quantification of spontaneous CNV (white asterisk). Scale bar = 500, 100 (1), and 25 (2) μm. **(D)** Representative analysis by immunofluorescence staining of isolectin B4 signal on RPE/choroid flatmounts in JR5558 mice 1 week after treatment with immunoglobulin G (IgG) control or anti–VEGF-A, anti–Ang-2, or bi-specific anti–Ang-2/VEGF-A antibodies. Scale bar = 500 μm. **(E)** Total area of isolectin B4 signal on RPE/choroid whole flatmounts in JR5558 mice 1 week after treatment with IgG control or anti–VEGF-A, anti–Ang-2, or bi-specific anti–Ang-2/VEGF-A antibodies (*n* = 20–24 flatmounts). **(F)** Representative infrared (IR) and fluorescein angiography (FA) images in JR5558 mice following treatment at day (D) 15 for CNV lesion leakage detection and quantification. **(G)** Lesion leakage area quantification in JR5558 mice 1 week after treatment following two weekly doses of IgG control or anti–VEGF-A, anti–Ang-2, or bi-specific anti–Ang-2/VEGF-A antibodies (*n* = 10 eyes). **(H)** Retinal vascular leakage detected in the vitreous of JR5558 mice, and corrected to plasma fluorescein concentration, using fluorescein fluorophotometry 1 week after treatment following two weekly doses of IgG control or anti–VEGF-A, anti–Ang-2, or bi-specific anti–Ang-2/VEGF-A antibodies (*n* = 7–8 eyes). Values are mean ± SD. One-way analysis of variance followed by Tukey’s multiple comparison test. **P < 0.01, ***P < 0.001, ****P < 0.0001. IHC, immunohistochemistry.

The short-term effects of dual Ang-2/VEGF-A inhibition on neovascularization, vascular leakage, and retinal inflammation were assessed in JR5558 mice (Figure 1A). Mice were given two intraperitoneal injections, separated by 1 week, of mouse cross-reactive antibodies against Ang-2, VEGF-A, or a bi-specific anti–Ang-2/VEGF-A antibody. Controls included IgG-injected and untreated mice. Leakage from sCNV lesions and blood-retinal barrier permeability were evaluated *in vivo* by FA and fluorophotometry, respectively, at 1 week after treatment. The extent of neovascularization was analyzed by immunohistochemistry using isolectin B4 as a vascular marker on RPE/choroid flatmounts to assess immediate treatment effects (Figure 1A). Following FA or RPE/choroid tissue image acquisition, fluorescein leakage or isolectin B4 signals were selected and quantified (Figure 1B).

Due to the variability of the number of lesions in the eyes, baseline sCNV lesion leakage area was assessed in each eye by FA, thereby allowing for equal distribution of number of lesions across experimental groups before treatment. As a result, there were no statistically significant differences in lesion leakage area number per eye (Supplementary Figure 1A) and lesion leakage area (Supplementary Figure 1B) at baseline before treatment administration between the untreated, IgG control, anti–VEGF-A, anti–Ang-2, and bi-specific anti–Ang-2/VEGF-A groups.

Evaluation of sCNV by isolectin B4 immunostaining on RPE/choroid flatmounts (Figure 1C) 1 week after treatment demonstrated that anti–VEGF-A, anti–Ang-2, and bi-specific anti–Ang-2/VEGF-A–treated mice all exhibited similar statistically significant reductions in total sCNV area compared with untreated and IgG control mice (Figures 1D, E). Bi-specific anti–Ang-2/VEGF-A–treated mice had the lowest mean sCNV area, although there were no statistically significant differences compared with anti–VEGF-A– or anti–Ang-2–treated mice. However, sCNV lesion leakage area was reduced only in anti–Ang-2/VEGF-A–treated mice compared with untreated or IgG-treated control mice (Figures 1F, G). The magnitude of reduction was most pronounced in bi-specific anti–Ang-2/VEGF-A–treated mice. There were no statistically significant between group differences in retinal vascular leakage detected in the vitreous 1 week after treatment; however, anti–VEGF-A, anti–Ang-2, and bi-specific anti–Ang-2/VEGF-A–treated mice had numerically less leakage than IgG control mice (Figure 1H). No phenotypic alteration or neovascular leakage was seen on IR or FA images from control mice without sCNV (Supplementary Figure 1C).

Together, these results confirm that dual Ang-2/VEGF-A inhibition may promote vascular stability by inhibiting neovascularization and subsequent neovascular leakage and are consistent with our previously reported findings (Regula et al., 2016; Foxton et al., 2019).

#### 3.1.2. Five weeks after treatment

**FIGURE 2:**
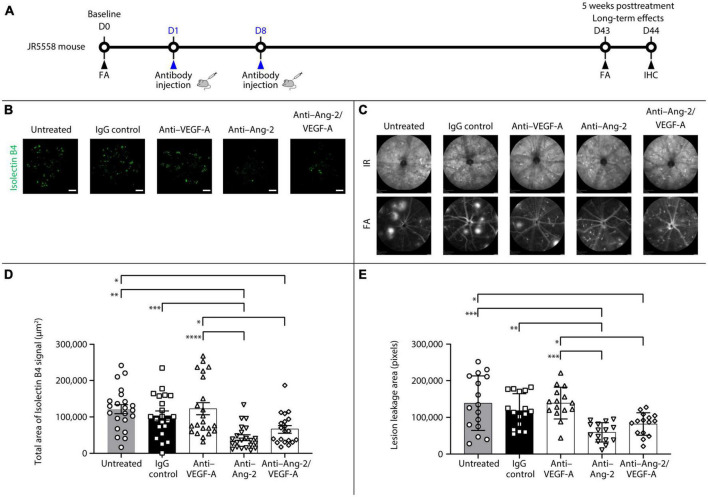
Dual angiopoietin-2 (Ang-2)/vascular endothelial growth factor-A (VEGF-A) inhibition causes sustained spontaneous choroidal neovascularization (CNV) and lesion leakage area reduction in JR5558 mice. **(A)** General experimental protocol used to assess long-term treatments effects on limiting vascular leakage, neovascularization, and inflammation in the JR5558 spontaneous CNV mouse model. **(B)** Representative analysis by immunofluorescence staining of isolectin B4 signal on retinal pigment epithelium (RPE)/choroid flatmounts in JR5558 mice 5 weeks after treatment with immunoglobulin G (IgG) control or anti–VEGF-A, anti–Ang-2, or bi-specific anti–Ang-2/VEGF-A antibodies. Scale bar = 500 μm. **(C)** Total area of isolectin B4 signal on RPE/choroid whole flatmounts in JR5558 mice 5 weeks after treatment with IgG control or anti–VEGF-A, anti–Ang-2, or bi-specific anti–Ang-2/VEGF-A antibodies (*n* = 20–22 flatmounts). **(D)** Representative infrared (IR) and fluorescein angiography (FA) images showing CNV leakage in JR5558 mice at day (D) 43 after treatment with IgG control or anti–VEGF-A, anti–Ang-2, or bi-specific anti–Ang-2/VEGF-A antibodies. **(E)** Lesion leakage area quantification in JR5558 mice 5 weeks after treatment with IgG control or anti–VEGF-A, anti–Ang-2, or bi-specific anti–Ang-2/VEGF-A antibodies (*n* = 17–18 eyes). Values are mean ± SD. One-way analysis of variance followed by Tukey’s multiple comparison test. *P < 0.05, **P < 0.01, ***P < 0.001, ****P < 0.0001. IHC, immunohistochemistry.

An additional set of experiments was performed to explore the potential long-term effects of bi-specific Ang-2/VEGF-A inhibition on vessel stabilization (Figure 2A).

After 5 weeks, anti–Ang-2– and bi-specific anti–Ang-2/VEGF-A–treated mice had statistically significantly reduced total sCNV area compared with untreated and anti–VEGF-A–treated mice. Only anti–Ang-2–treated mice had a statistically significant reduction in isolectin B4 signal compared with IgG control mice (Figures 2B, C). Moreover, anti–Ang-2– and bi-specific anti–Ang-2/VEGF-A–treated mice had statistically significantly decreased sCNV lesion leakage area compared with untreated control and anti–VEGF-A–treated mice. Only anti–Ang-2–treated mice had reduced sCNV area compared with IgG control mice (Figures 2D, E).

Taken together, the results obtained 5 weeks after treatment demonstrated that Ang-2 and dual Ang-2/VEGF-A inhibition were superior to VEGF-A inhibition in promoting vascular stability by reducing neovascularization and neovascular leakage, further suggesting the prolonged beneficial effect of inhibiting Ang-2 and VEGF over anti-VEGF alone may be driven by Ang-2 inhibition.

### 3.2. Dual Ang-2/VEGF-A inhibition reduces subretinal Iba1^+^, CD11b^+^, and CD45^+^ cell infiltration in JR5558 mice

#### 3.2.1. One week after treatment

The short-term effects of dual Ang-2/VEGF-A inhibition on subretinal infiltration of Iba1, CD11b, and CD45 immune cells were evaluated by immunohistochemistry on RPE/choroid flatmounts 1 week after treatment in JR5558 mice. Iba1 is a microglia-/macrophage-specific marker (Sousa et al., 2017), and CD11b is expressed on the surface of microglia and all myeloid leukocytes, including monocytes, macrophages, granulocytes (including neutrophils), and natural killer cells. CD45 is known as the common leukocyte marker and is expressed by microglia, myeloid leukocytes, and lymphocytes (Jurga et al., 2020).

**FIGURE 3:**
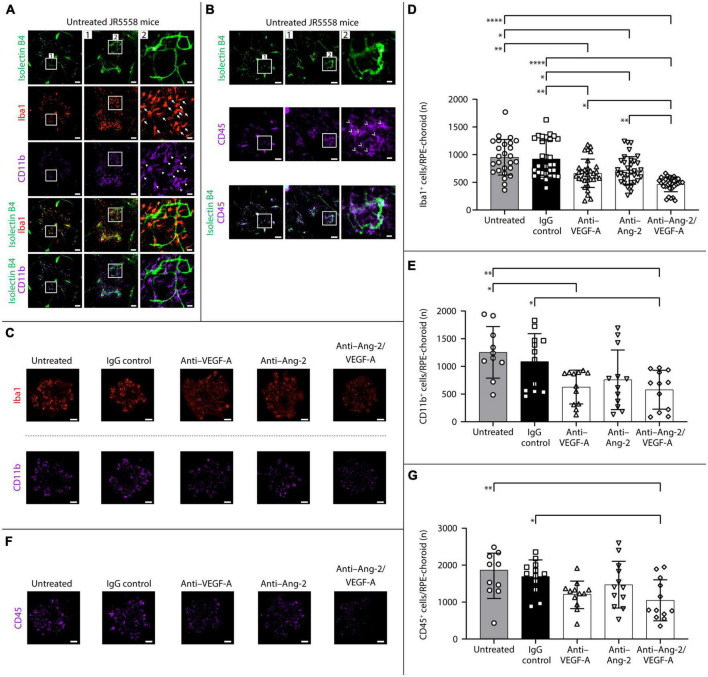
Dual angiopoietin-2 (Ang-2)/vascular endothelial growth factor-A (VEGF-A) inhibition reduces subretinal ionized calcium binding adapter molecule 1 (Iba1^+^), CD11b^+^, and CD45^+^ cell infiltration in JR5558 mice vs. anti-VEGF alone. **(A,B)** Representative isolectin B4 (white asterisk), Iba1, CD11b, and CD45 immunostaining images detected on retinal pigment epithelium (RPE)/choroid flatmounts from untreated JR5558 mice, allowing visualization and quantification of subretinal Iba1^+^ (white arrows), CD11b^+^ (white triangles), and CD45^+^ (white arrowhead) immune cells infiltration. Scale bar = 500, 100 (1), and 25 (2) μm. **(C)** Representative analysis by immunofluorescence staining of Iba1 or CD11b on RPE/choroid flatmounts in JR5558 mice 1 week after treatment with immunoglobulin G (IgG) control or anti–VEGF-A, anti–Ang-2, or bi-specific anti–Ang-2/VEGF-A antibodies. Scale bar = 500 μm. **(D,E)** Total number of Iba1^+^ **(D)** or CD11b^+^ **(E)** inflammatory cells around choroidal neovascularization (CNV) lesions on RPE/choroid whole flatmounts in JR5558 mice 1 week after treatment with IgG control or anti–VEGF-A, anti–Ang-2, or bi-specific anti–Ang-2/VEGF-A antibodies [*n* = 26–34 flatmounts for panel **(D)** and *n* = 10–12 flatmounts for panel **(E)**]. **(F)** Representative immunofluorescence staining of CD45 on RPE/choroid flatmounts in JR5558 mice 1 week after treatment with IgG control, anti–VEGF-A, anti–Ang-2, or bi-specific anti–Ang-2/VEGF-A antibodies. Scale bar = 500 μm. **(G)** Total number of CD45^+^ inflammatory cells around CNV lesions on RPE/choroid whole flatmounts 1 week after treatment with IgG control or anti–VEGF-A, anti–Ang-2, or bi-specific anti–Ang-2/VEGF-A antibodies (*n* = 10–12 flatmounts). Values are mean ± SD. One-way analysis of variance followed by Tukey’s multiple comparison test. *P < 0.05, **P < 0.01, ****P < 0.0001.

Representative immunostaining on RPE/choroid flatmounts from untreated JR5558 mice revealed Iba1^+^, CD11b^+^, and CD45^+^ immune cells were located mainly around sCNV lesions (Figures 3A, B). In control mice without sCNV, virtually no immune cells were detected (Supplementary Figure 1C). At 1 week after treatment, the number of Iba1^+^ microglia/macrophages on RPE/choroid and around sCNV lesions was statistically significantly reduced in anti–VEGF-A–, anti–Ang-2–, and bi-specific anti–Ang-2/VEGF-A–treated mice compared with untreated or IgG control mice. Moreover, bi-specific anti–Ang-2/VEGF-A–treated mice had a further decreased number of Iba1^+^ cells compared with anti–VEGF-A– or anti–Ang-2–treated mice (Figures 3C, D). The number of CD11b^+^ myeloid leukocytes on RPE/choroid and around sCNV lesions was statistically significantly decreased in both anti-VEGF–treated and bi-specific anti–Ang-2/VEGF-A–treated mice compared with untreated control mice, whereas only bi-specific anti–Ang-2/VEGF-A–treated mice exhibited a statistically significant decrease compared with IgG control mice (Figures 3C, E). Only bi-specific anti–Ang-2/VEGF-A–treated mice exhibited a statistically significant decrease in number of CD45^+^ cells compared with both untreated and IgG control mice (Figures 3F, G).

Overall, the Iba1, CD11b, and CD45 immunostaining data indicate that dual Ang-2/VEGF-A inhibition decreases immune cell accumulation associated with sCNV lesions, statistically significantly more so than VEGF-A or Ang-2 inhibition alone for Iba1 + accumulation.

#### 3.2.2. Five weeks after treatment

An additional set of experiments was performed to explore potential long-term effects of bi-specific Ang-2/VEGF-A inhibition on subretinal infiltration of Iba1^+^, CD11b^+^, and CD45^+^ immune cells.

**FIGURE 4:**
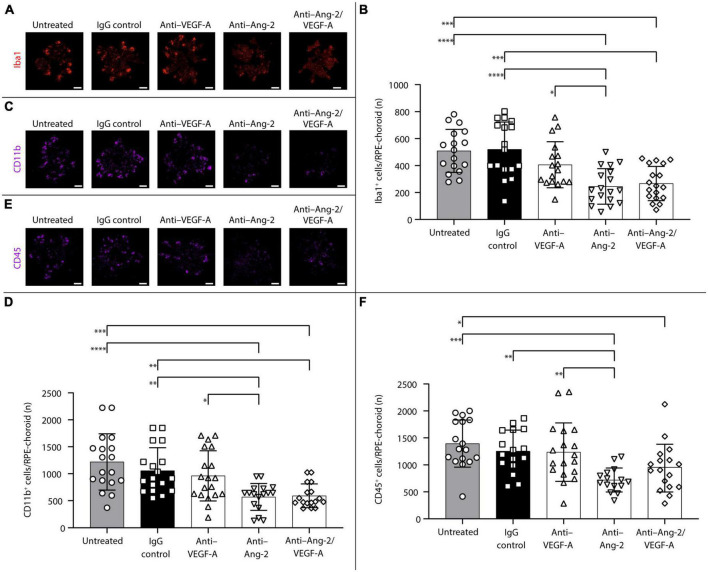
Dual inhibition of angiopoietin-2 (Ang-2)/vascular endothelial growth factor-A (VEGF-A) causes sustained reduction in subretinal ionized calcium binding adapter molecule 1 (Iba1^+^), CD11b^+^, and CD45^+^ inflammatory cells infiltration in JR5558 mice. **(A,C,E)** Representative analysis by immunofluorescence staining of Iba1 **(A)**, CD11b **(C)**, or CD45 **(E)** on retinal pigment epithelium (RPE)/choroid flatmounts in JR5558 mice 5 weeks after treatment with immunoglobulin G (IgG) control or anti–VEGF-A, anti–Ang-2, or bi-specific anti–Ang-2/VEGF-A antibodies. Scale bar = 500 μm. **(B,D,F)** Total number of Iba1^+^ **(B)**, CD11b^+^, **(D)** or CD45^+^ **(F)** inflammatory cells around choroidal neovascularization lesions on RPE/choroid whole flatmounts in JR5558 mice 5 weeks after treatment with IgG control or anti–VEGF-A, anti–Ang-2, or bi-specific anti–Ang-2/VEGF-A antibodies [*n* = 17–18 flatmounts for panel **(B)**, *n* = 16–18 for panel **(D)**, and *n* = 15–18 flatmounts for panel **(F)**]. Values are mean ± SD. One-way analysis of variance followed by Tukey’s multiple comparison test. *P < 0.05, **P < 0.01, ***P < 0.001, ****P < 0.0001.

After 5 weeks, anti–Ang-2– and bi-specific anti–Ang-2/VEGF-A–treated mice exhibited statistically significant decreases in the number of Iba1^+^ (Figures 4A, B) and CD11b^+^ (Figures 4C, D) cells on RPE/choroid and around sCNV lesions compared with control group mice. Anti–Ang-2–treated mice also exhibited a statistically significant decrease in the number of CD45^+^ cells compared with control group mice (Figures 4E, F). Decreases in all immune cell counts were also statistically significantly more pronounced in anti–Ang-2–treated mice compared with anti–VEGF-A–treated mice. There were no statistically significant differences in the number of Iba1^+^ cells between bi-specific anti–Ang-2/VEGF-A–treated mice and anti–Ang-2– or anti–VEGF–treated mice.

Taken together, the results obtained 5 weeks after treatment demonstrated that Ang-2 and dual Ang-2/VEGF-A inhibition were superior to VEGF-A inhibition in reducing inflammation, suggesting that the sustained anti-inflammatory effect observed with dual Ang-2/VEGF-A inhibition over anti-VEGF alone may be driven by Ang-2 inhibition.

### 3.3. Dual Ang-2/VEGF-A inhibition prevents I/R injury–induced retinal permeability and cell death

**FIGURE 5:**
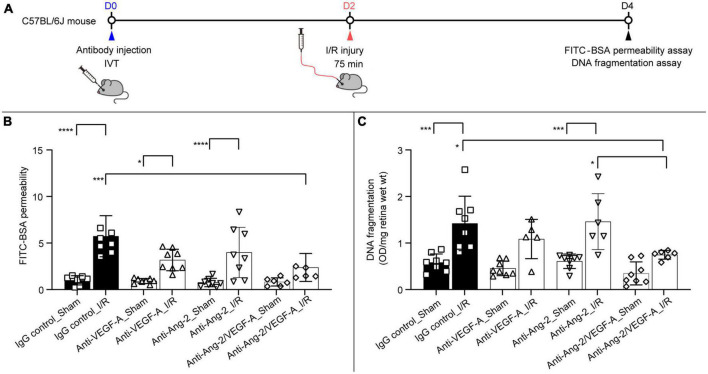
Dual angiopoietin-2 (Ang-2)/vascular endothelial growth factor-A (VEGF-A) inhibition prevents ischemia/reperfusion (I/R) injury–induced retinal permeability and cell death. **(A)** General experimental protocol used to assess treatment effects on limiting vascular permeability and cell death in the retinal I/R injury mouse model. **(B,C)** Retinal fluorescein isothiocyanate–labeled bovine serum albumin (FITC-BSA) permeability **(B)** and retinal apoptotic DNA fragmentation **(C)** 2 days after I/R injury following intravitreal injection of immunoglobulin G (IgG) control, anti–VEGF-A, anti–Ang-2, or bi-specific anti–Ang-2/VEGF-A antibodies (*n* = 5–8 eyes) 4 days before analysis. Values are mean ± SD. One-way analysis of variance followed by Tukey’s multiple comparison test. *P < 0.05, ***P < 0.001, ****P < 0.0001. D, day; IVT, intravitreal.

To further explore the protective effects of dual Ang-2/VEGF-A inhibition, the retinal I/R mouse model (Muthusamy et al., 2014) was used to evaluate changes in vascular permeability and cell death following retinal injury. The mouse retinal I/R model is characterized by progressive neurodegeneration of the inner retina and immediate and sustained breakdown of the inner blood-retina barrier (Abcouwer et al., 2021). Mice were given intravitreal injections of anti–VEGF-A, anti–Ang-2, and bi-specific anti–Ang-2/VEGF-A antibodies 2 days before I/R injury (Figure 5A). I/R comprised ischemia for 75 min followed by natural reperfusion to induce retinal injury. Vascular permeability was measured by evaluating FITC-BSA extravasation 48 h after reperfusion.

Bi-specific anti–Ang-2/VEGF-A–treated mice had statistically significantly reduced retinal vascular permeability (by 64%) compared with IgG control–treated mice (Figure 5B). There was no statistically significant difference between IgG control and anti–VEGF-A– or anti–Ang-2–treated mice (Figure 5B). Consistent with these findings, assessment of neuronal cell death by DNA fragmentation demonstrated that cell death was statistically significantly reduced (by 47%) in bi-specific anti–Ang-2/VEGF-A–treated mice compared with IgG control mice (Figure 5C). In contrast, there was no evidence of protection in monotherapy anti–VEGF-A– or anti–Ang-2–treated mice (Figure 5C).

These results suggest that intravitreal delivery of antibodies affording dual Ang-2/VEGF-A inhibition is more effective than antibodies causing Ang-2 or VEGF-A inhibition alone in preventing both vascular permeability and neurodegeneration following retinal I/R injury.

## 4. Discussion

Phase 3 clinical trials in patients with DME or nAMD have demonstrated that dual Ang-2 and VEGF-A pathway inhibition with faricimab improves and maintains vision, with strong durability, and improves anatomic outcomes compared with VEGF-A pathway inhibition alone. However, the underlying individual contributions of Ang-2 and VEGF-A in mediating these outcomes is unclear. Here, we have described the results of preclinical studies that aimed to delineate the longer-term (5-week) effects of Ang-2 and Ang-2/VEGF-A inhibition on vascular permeability, inflammation, and neurodegeneration in mouse models of retinal neovascularization and I/R. Of note, our findings highlight that Ang-2 plays an important underlying role in the extended efficacy and durability effects observed here.

We used the JR5558 mouse model of sCNV to evaluate the effects of dual Ang-2/VEGF-A inhibition, Ang-2 inhibition alone, and VEGF-A inhibition alone on neovascularization, vascular leakage, and subretinal immune cell infiltration 1 and 5 weeks after treatment. Dual Ang-2/VEGF-A inhibition consistently reduced both sCNV and lesion leakage area, as well subretinal immune cell infiltration compared with control treatment, whereas the effects of Ang-2 and VEGF-A inhibition varied. Notably, we found that after 1 week, Ang-2, VEGF-A, and dual Ang-2/VEGF-A inhibition all reduced total sCNV area; reductions were greatest with dual Ang-2/VEGF-A pathway inhibition. Furthermore, only dual Ang-2/VEGF-A inhibition decreased lesion leakage area. Interestingly, after 5 weeks, only Ang-2 and dual Ang-2/VEGF-A inhibition maintained their CNV leakage-reducing effects. Similarly, after 1 week, Ang-2, VEGF-A, and dual Ang-2/VEGF-A inhibition all reduced immune cells (Iba1^+^, CD11b^+^, and CD45^+^) accumulation at the lesions, whereas only Ang-2 and dual Ang-2/VEGF-A inhibition, but not VEGF-A inhibition, reduced immune cells accumulation after 5 weeks. Taken together, these findings not only suggest that dual Ang-2/VEGF-A inhibition may promote vascular stability by decreasing neovascular leakage and neovascularization, but also that Ang-2 inhibition plays an important underlying role, particularly when considering the more prolonged (5-week) effects. Further, these findings suggest that Ang-2 inhibition may play an important role in mediating the strong durability and anatomic improvement observed with faricimab in clinical trials (Heier et al., 2022; Khanani et al., 2022; Lim et al., 2022; Wykoff et al., 2022).

We have previously reported that I/R injury causes a rapid and sustained breakdown of the inner blood-retina barrier (Abcouwer et al., 2021). The rapidity of modification and disorganization of tight junctions at the endothelial cell borders suggests that I/R may have a direct effect on the retinal vasculature. We also found that this permeability response coincides with VEGF receptor-2 phosphorylation in the mouse and that post-I/R permeability can be reduced by anti-VEGF pre-treatment in the rat (Abcouwer et al., 2010; Muthusamy et al., 2014). The results described herein show that intravitreal delivery of a dual Ang-2/VEGF-A–inhibiting antibody was superior to antibodies inhibiting Ang-2 or VEGF alone in preventing the vascular permeability response to I/R injury. This finding suggests that both Ang-2 and VEGF signaling contribute to this response. Surprisingly, dual Ang-2/VEGF-A inhibition, but not Ang-2 or VEGF-A inhibition alone, also demonstrated neuroprotective effects as indicated by a decreased rate of neuronal cell death. Further research is needed to determine whether these protective effects are the result of direct or indirect effects secondary to inhibition of vascular leakage. However, because the antibody delivery was ocular in the I/R injury model, rather than systemic as in the sCNV model, the results suggest that the effects on retinal pathology are due to dual Ang-2/VEGF-A inhibition in the retina, not due to possible systemic effects on immune cells.

In conclusion, our findings suggest that targeting both Ang-2 and VEGF-A can provide additional protective benefits in models of ocular neovascularization and pathological retinal vascular permeability. This seems to be due to the complementary effects of inhibiting Ang-2 and VEGF-A on vascular stability. Importantly, our findings also suggest that Ang-2 inhibition may be particularly important in mediating the extended effects of dual Ang-2/VEGF-A inhibition. These preclinical findings are consistent with the extended durability of faricimab observed in phase 3 clinical trials and the greater reductions in anatomic parameters observed with faricimab compared with anti-VEGF therapies (Cheung et al., 2022; Heier et al., 2022; Khanani et al., 2022; Lim et al., 2022; Wykoff et al., 2022). In the mouse models, dual Ang-2/VEGF-A inhibition also showed increased efficacy against immune cell attraction to sCNV lesions and neurodegeneration after retinal I/R injury. Further studies are needed to determine if these effects are secondary to vascular stabilization or due to direct anti-inflammatory and neuroprotective effects of inhibiting Ang-2.

## Data availability statement

The raw data supporting the conclusions of this article will be made available by the authors, without undue reservation.

## Ethics statement

Animal maintenance and experimental procedures were approved by the Federal Food Safety and Veterinary Office of Switzerland (reference BS-2734) and were conducted in strict adherence to the Swiss federal ordinance on animal protection and welfare, as well as according to the rules of the Association for Research in Vision and Ophthalmology Statement for the Use of Animals in Ophthalmic and Vision Research Guidelines (European Directive 86/609/EEC) and the Roche Ethics Committee on Animal Welfare (JR5558 sCNV mouse model) or with approval of the University of Michigan Institutional Animal Care and Use Committee (retinal I/R injury mouse model).

## Author contributions

All authors had full access to all the data in the study, took responsibility for the integrity of the data and the accuracy of the data analysis, and contributed to the planning, conduct, and reporting of the work described in the article.

## References

[B1] Abcouwer S. F. Lin C. M. Wolpert E. B. Shanmugam S. Schaefer E. W. Freeman W. M. (2010). Effects of ischemic preconditioning and bevacizumab on apoptosis and vascular permeability following retinal ischemia-reperfusion injury. *Invest. Ophthalmol. Vis. Sci.* 51 5920–5933. 10.1167/iovs.10-5264 20554620

[B2] Abcouwer S. F. Shanmugam S. Muthusamy A. Lin C. M. Kong D. Hager H. (2021). Inflammatory resolution and vascular barrier restoration after retinal ischemia reperfusion injury. *J. Neuroinflamm.* 18:186. 10.1186/s12974-021-02237-5 34446062 PMC8394696

[B3] Adamis A. P. Brittain C. J. Dandekar A. Hopkins J. J. (2020). Building on the success of anti-vascular endothelial growth factor therapy: A vision for the next decade. *Eye* 34 1966–1972. 10.1038/s41433-020-0895-z 32541890 PMC7784857

[B4] Antonetti D. A. Silva P. S. Stitt A. W. (2021). Current understanding of the molecular and cellular pathology of diabetic retinopathy. *Nat. Rev. Endocrinol.* 17 195–206. 10.1038/s41574-020-00451-4 33469209 PMC9053333

[B5] Busbee B. G. Ho A. C. Brown D. M. Heier J. S. Suner I. J. Li Z. (2013). Twelve-month efficacy and safety of 0.5 mg or 2.0 mg ranibizumab in patients with subfoveal neovascular age-related macular degeneration. *Ophthalmology* 120 1046–1056. 10.1016/j.ophtha.2012.10.014 23352196

[B6] Campochiaro P. A. (2013). Ocular neovascularization. *J. Mol. Med.* 91 311–321. 10.1007/s00109-013-0993-5 23329331 PMC3584193

[B7] Campochiaro P. A. (2015). Molecular pathogenesis of retinal and choroidal vascular diseases. *Prog. Retin. Eye Res.* 49 67–81. 10.1016/j.preteyeres.2015.06.002 26113211 PMC4651818

[B8] Chakravarthy U. Bailey C. Brown D. Campochiaro P. Chittum M. Csaky K. (2017). Phase I trial of anti-vascular endothelial growth factor/anti-angiopoietin 2 bispecific antibody RG7716 for neovascular age-related macular degeneration. *Ophthalmol. Retina* 1 474–485. 10.1016/j.oret.2017.03.003 31047438

[B9] Cheung C. M. Holz F. G. Khanani A. M. Kotecha A. Patel A. Willis J. R. (2022). “Faricimab in neovascular age-related macular degeneration: Year 2 efficacy, safety and durability results from the phase 3 TENAYA and LUCERNE trials,” in *Paper presented at Congress of the Asia-Pacific Vitreo-retina Society, November 18–20*, (Taipei: Congress of the Asia-Pacific Vitreo-retina Society).

[B10] Ciulla T. A. Hussain R. M. Pollack J. S. Williams D. F. (2020). Visual acuity outcomes and anti-vascular endothelial growth factor therapy intensity in neovascular age-related macular degeneration patients: A real-world analysis of 49 485 eyes. *Ophthalmol. Retina* 4 19–30. 10.1016/j.oret.2019.05.017 31324588

[B11] Ciulla T. A. Pollack J. S. Williams D. F. (2021). Visual acuity outcomes and anti-VEGF therapy intensity in diabetic macular oedema: A real-world analysis of 28 658 patient eyes. *Br. J. Ophthalmol.* 105 216–221. 10.1136/bjophthalmol-2020-315933 32265201 PMC7848066

[B12] Felcht M. Luck R. Schering A. Seidel P. Srivastava K. Hu J. (2012). Angiopoietin-2 differentially regulates angiogenesis through TIE2 and integrin signaling. *J. Clin. Invest.* 122 1991–2005. 10.1172/JCI58832 22585576 PMC3366398

[B13] Flaxman S. R. Bourne R. R. A. Resnikoff S. Ackland P. Braithwaite T. Cicinelli M. V. (2017). Global causes of blindness and distance vision impairment 1990-2020: A systematic review and meta-analysis. *Lancet Glob. Health* 5 e1221–e1234. 10.1016/S2214-109X(17)30393-5 29032195

[B14] Foxton R. H. Uhles S. Grüner S. Revelant F. Ullmer C. (2019). Efficacy of simultaneous VEGF-A/ANG-2 neutralization in suppressing spontaneous choroidal neovascularization. *EMBO Mol. Med.* 11:e10204. 10.15252/emmm.201810204 31040126 PMC6505683

[B15] Grossniklaus H. E. Ling J. X. Wallace T. M. Dithmar S. Lawson D. H. Cohen C. (2002). Macrophage and retinal pigment epithelium expression of angiogenic cytokines in choroidal neovascularization. *Mol. Vis.* 8 119–126. 11979237

[B16] Hakanpaa L. Sipila T. Leppanen V. M. Gautam P. Nurmi H. Jacquemet G. (2015). Endothelial destabilization by angiopoietin-2 via integrin β1 activation. *Nat. Commun.* 6:5962. 10.1038/ncomms6962 25635707 PMC4316742

[B17] Hartsock M. J. Cho H. Wu L. Chen W. J. Gong J. Duh E. J. (2016). A mouse model of retinal ischemia-reperfusion injury through elevation of intraocular pressure. *J. Vis. Exp.* 14:54065. 10.3791/54065 27501124 PMC5091361

[B18] Hasegawa E. Sweigard H. Husain D. Olivares A. M. Chang B. Smith K. E. (2014). Characterization of a spontaneous retinal neovascular mouse model. *PLoS One* 9:e106507. 10.1371/journal.pone.0106507 25188381 PMC4154693

[B19] Heier J. S. Khanani A. M. Quezada Ruiz C. Basu K. Ferrone P. J. Brittain C. (2022). Efficacy, durability, and safety of intravitreal faricimab up to every 16 weeks for neovascular age-related macular degeneration (TENAYA and LUCERNE): Two randomised, double-masked, phase 3, non-inferiority trials. *Lancet* 399 729–740. 10.1016/S0140-6736(22)00010-1 35085502

[B20] Joussen A. M. Ricci F. Paris L. P. Korn C. Quezada-Ruiz C. Zarbin M. (2021). Angiopoietin/Tie2 signalling and its role in retinal and choroidal vascular diseases: A review of preclinical data. *Eye* 35 1305–1316. 10.1038/s41433-020-01377-x 33564135 PMC8182896

[B21] Jurga A. M. Paleczna M. Kuter K. Z. (2020). Overview of general and discriminating markers of differential microglia phenotypes. *Front. Cell Neurosci.* 14:198. 10.3389/fncel.2020.00198 32848611 PMC7424058

[B22] Khanani A. M. Demtriades A.-M. Kotecha A. Silverman D. Swaminathan B. Patel V. (2022). “Faricimab in neovascular age-related macular degeneration: Year 2 efficacy, safety, and durability results from the phase 3 TENAYA and LUCERNE trials,” in *Paper presented at American society of retina specialists annual meeting*, (New York, NY: ASRS).

[B23] Khanani A. M. Patel S. S. Ferrone P. J. Osborne A. Sahni J. Grzeschik S. (2020). Efficacy of every four monthly and quarterly dosing of faricimab vs ranibizumab in neovascular age-related macular degeneration: The STAIRWAY phase 2 randomized clinical trial. *JAMA Ophthalmol.* 138 964–972. 10.1001/jamaophthalmol.2020.2699 32729897 PMC7489851

[B24] Laouri M. Chen E. Looman M. Gallagher M. (2011). The burden of disease of retinal vein occlusion: Review of the literature. *Eye* 25 981–988. 10.1038/eye.2011.92 21546916 PMC3178209

[B25] Lim J. I Wells J. A. Eichenbaum D. A. Danzig C. J. Asik K. Haskova Z. (2022). Efficacy, durability, and safety of faricimab in diabetic macular edema: 2-year results from the phase 3 YOSEMITE and RHINE trials. *Investig. Ophthalmol. Vis. Sci.* 63:3850.

[B26] Martin D. F. Maguire M. G. Fine S. L. Ying G. S. Jaffe G. J. Grunwald J. E. (2012). Ranibizumab and bevacizumab for treatment of neovascular age-related macular degeneration: Two-year results. *Ophthalmology* 119 1388–1398. 10.1016/j.ophtha.2012.03.053 22555112 PMC3389193

[B27] Muthusamy A. Lin C. M. Shanmugam S. Lindner H. M. Abcouwer S. F. Antonetti D. A. (2014). Ischemia-reperfusion injury induces occludin phosphorylation/ubiquitination and retinal vascular permeability in a VEGFR-2-dependent manner. *J. Cereb. Blood Flow Metab.* 34 522–531. 10.1038/jcbfm.2013.230 24398936 PMC3948134

[B28] Nagai N. Lundh von Leithner P. Izumi-Nagai K. Hosking B. Chang B. Hurd R. (2014). Spontaneous CNV in a novel mutant mouse is associated with early VEGF-A-driven angiogenesis and late-stage focal edema, neural cell loss, and dysfunction. *Invest. Ophthalmol. Vis. Sci.* 55 3709–3719. 10.1167/iovs.14-13989 24845632 PMC4059080

[B29] Nguyen Q. D. Brown D. M. Marcus D. M. Boyer D. S. Patel S. Feiner L. (2012). Ranibizumab for diabetic macular edema: Results from 2 phase III randomized trials: RISE and RIDE. *Ophthalmology* 119 789–801. 10.1016/j.ophtha.2011.12.039 22330964

[B30] Regula J. T. Lundh von Leithner P. Foxton R. Barathi V. A. Cheung C. M. Bo Tun S. B. (2016). Targeting key angiogenic pathways with a bispecific CrossMAb optimized for neovascular eye diseases. *EMBO Mol. Med.* 8 1265–1288. 10.15252/emmm.201505889 27742718 PMC5090659

[B31] Regula J. T. Lundh von Leithner P. Foxton R. Barathi V. A. Chui Ming G. C. Tun S. B. B. (2019). Targeting key angiogenic pathways with a bispecific CrossMAb optimized for neovascular eye diseases. *EMBO Mol. Med.* 11:e10666. 10.15252/emmm.201910666 31040127 PMC6505574

[B32] Rossato F. A. Su Y. Mackey A. Ng Y. S. E. (2020). Fibrotic changes and endothelial-to-mesenchymal transition promoted by VEGFR2 antagonism alter the therapeutic effects of VEGFA pathway blockage in a mouse model of choroidal neovascularization. *Cells* 9:2057. 10.3390/cells9092057 32917003 PMC7563259

[B33] Rübsam A. Parikh S. Fort P. E. (2018). Role of inflammation in diabetic retinopathy. *Int. J. Mol. Sci.* 19:942. 10.3390/ijms19040942 29565290 PMC5979417

[B34] Saharinen P. Eklund L. Alitalo K. (2017). Therapeutic targeting of the angiopoietin-TIE pathway. *Nat. Rev. Drug Discov.* 16 635–661. 10.1038/nrd.2016.278 28529319

[B35] Sahni J. Dugel P. U. Patel S. S. Chittum M. E. Berger B. Del Valle Rubido M. (2020). Safety and efficacy of different doses and regimens of faricimab vs ranibizumab in neovascular age-related macular degeneration: The AVENUE phase 2 randomized clinical trial. *JAMA Ophthalmol.* 138 955–963. 10.1001/jamaophthalmol.2020.2685 32729888 PMC7393587

[B36] Sahni J. Patel S. S. Dugel P. U. Khanani A. M. Jhaveri C. D. Wykoff C. C. (2019). Simultaneous inhibition of angiopoietin-2 and vascular endothelial growth factor-a with faricimab in diabetic macular edema: BOULEVARD phase 2 randomized trial. *Ophthalmology* 126 1155–1170. 10.1016/j.ophtha.2019.03.023 30905643

[B37] Sousa C. Biber K. Michelucci A. (2017). Cellular and molecular characterization of microglia: A unique immune cell population. *Front. Immunol.* 8:198. 10.3389/fimmu.2017.00198 28303137 PMC5332364

[B38] Spooner K. Fraser-Bell S. Hong T. Chang A. A. (2019). Five-year outcomes of retinal vein occlusion treated with vascular endothelial growth factor inhibitors. *BMJ Open Ophthalmol.* 4:e000249. 10.1136/bmjophth-2018-000249 30997407 PMC6440600

[B39] Titchenell P. M. Antonetti D. A. (2013). Using the past to inform the future: Anti-VEGF therapy as a road map to develop novel therapies for diabetic retinopathy. *Diabetes* 62 1808–1815. 10.2337/db12-1744 23704522 PMC3661651

[B40] Varano M. Eter N. Winyard S. Wittrup-Jensen K. U. Navarro R. Heraghty J. (2015). Current barriers to treatment for wet age-related macular degeneration (wAMD): Findings from the wAMD patient and caregiver survey. *Clin. Ophthalmol.* 9 2243–2250. 10.2147/OPTH.S92548 26664038 PMC4671808

[B41] Wells J. A. Glassman A. R. Ayala A. R. Jampol L. M. Bressler N. M. Bressler S. B. (2016). Aflibercept, bevacizumab, or ranibizumab for diabetic macular edema: Two-year results from a comparative effectiveness randomized clinical trial. *Ophthalmology* 123 1351–1359. 10.1016/j.ophtha.2016.02.022 26935357 PMC4877252

[B42] Wykoff C. C. Abreu F. Adamis A. P. Basu K. Eichenbaum D. A. Haskova Z. (2022). Efficacy, durability, and safety of intravitreal faricimab with extended dosing up to every 16 weeks in patients with diabetic macular oedema (YOSEMITE and RHINE): Two randomised, double-masked, phase 3 trials. *Lancet* 399 741–755. 10.1016/S0140-6736(22)00018-6 35085503

