# Supplementary material for: Delineating effects of angiopoietin-2 inhibition on vascular permeability and inflammation in models of retinal neovascularization and ischemia/reperfusion
Source: Front Cell Neurosci. 2023 Jun 12;17:1192464. doi: 10.3389/fncel.2023.1192464 (PMC10291265; doi:10.3389/fncel.2023.1192464)

SUPPLEMENTAL FIGURE 1

Phenotypic analysis in untreated JR5558 mice and control animals without spontaneous choroidal neovascularization (CNV). (**A, B**) Lesion leakage area number per eye (**A**) and lesion leakage area (**B**) quantification in JR5558 mice at baseline before treatment with immunoglobulin G (IgG) control or anti–VEGF-A, anti–angiopoietin-2 (Ang-2), or bi-specific anti–Ang-2/vascular endothelial growth factor-A (VEGF-A) antibodies (*n* = 18–20 eyes for **A** and *n* = 9–10 eyes for **B**). (**C**) Representative infrared (IR) and fluorescein angiography (FA) images and isolectin B4, ionized calcium binding adapter molecule 1 (Iba1), and CD11b immunostainings detected on retinal pigment epithelium/choroid flatmounts from control mice without spontaneous CNV. Scale bar = 500 µm. Values are mean ± SD. D, day.

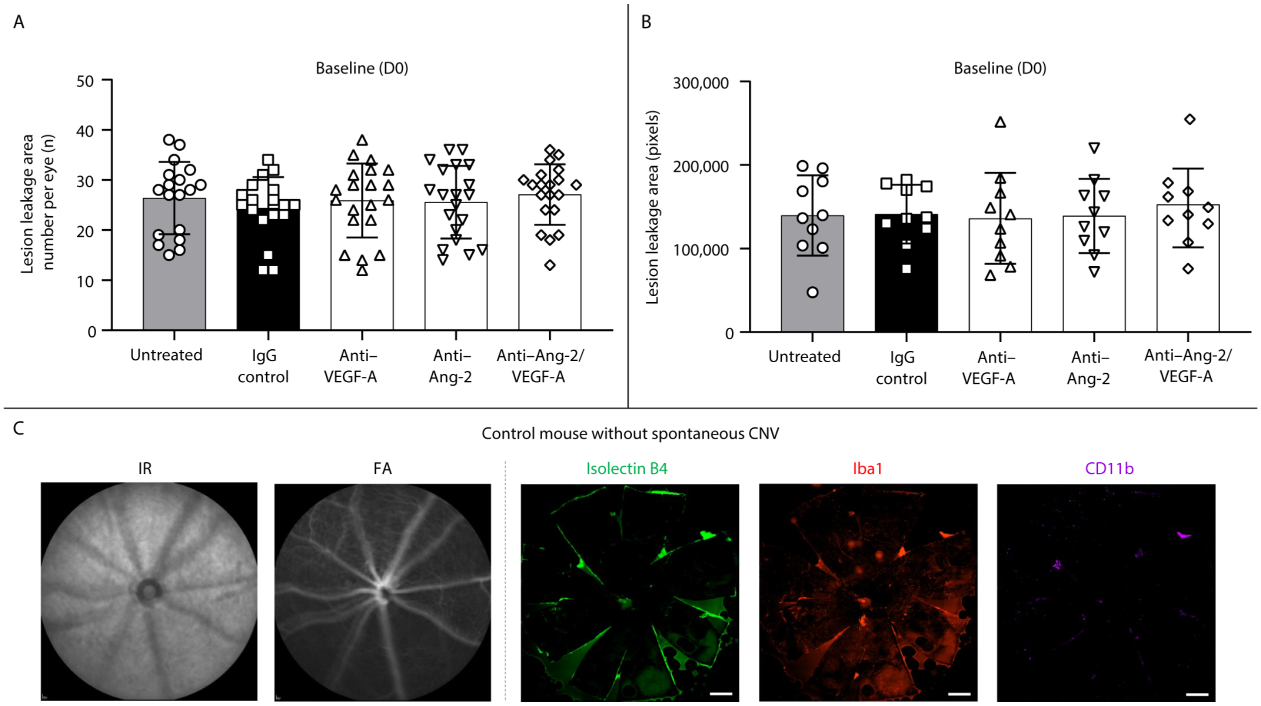

Supplement: Supplementary file 1 [file Image_1.PDF]
